# No vagina, one vagina, or multiple vaginae? An integrative study of *Pseudaxine trachuri* (Monogenea, Gastrocotylidae) leads to a better understanding of the systematics of *Pseudaxine* and related genera

**DOI:** 10.1051/parasite/2020046

**Published:** 2020-08-18

**Authors:** Chahinez Bouguerche, Fadila Tazerouti, Delphine Gey, Jean-Lou Justine

**Affiliations:** 1 Université des Sciences et de la Technologie Houari Boumediene, Faculté des Sciences Biologiques, Laboratoire de Biodiversité et Environnement: Interactions – Génomes BP 32, El Alia Bab Ezzouar 16111 Alger Algérie; 2 Service de Systématique Moléculaire, UMS 2700 CNRS, Muséum National d’Histoire Naturelle, Sorbonne Universités 43 Rue Cuvier, CP 26 75231 Paris Cedex 05 France; 3 UMR7245 MCAM, Muséum National d’Histoire Naturelle 61, Rue Buffon, CP52 75231 Paris Cedex 05 France; 4 Institut Systématique Évolution Biodiversité (ISYEB), Muséum National d’Histoire Naturelle, CNRS, Sorbonne Université, EPHE, Université des Antilles 57 Rue Cuvier, CP 51 75231 Paris Cedex 05 France

**Keywords:** Monogenea, Gastrocotylidae, *Pseudaxine*, *Pseudaxinoides*, *Allogastrocotyle*, Vagina, Mediterranean Sea, Molecular barcoding

## Abstract

The presence/absence and number of vaginae is a major characteristic for the systematics of the Monogenea. Three gastrocotylid genera share similar morphology and anatomy but are distinguished by this character: *Pseudaxine* Parona & Perugia, 1890 has no vagina, *Allogastrocotyle* Nasir & Fuentes Zambrano, 1983 has two vaginae, and *Pseudaxinoides* Lebedev, 1968 has multiple vaginae. In the course of a study of *Pseudaxine trachuri* Parona & Perugia 1890, we found specimens with structures resembling “multiple vaginae”; we compared them with specimens without vaginae in terms of both morphology and molecular characterisitics (COI barcode), and found that they belonged to the same species. We also investigated the male copulatory organ (MCO) of this species, the accuracy of the original description of which is known to be a matter of debate. We found that the genital atrium is armed with 12 hooks arranged as a single circle and a central hollow stylet which is probably involved in traumatic insemination. We redescribed *Pseudaxine trachuri* based on newly collected specimens from off the coast of Algeria and Museum specimens from off France. Specimens from the type-host, *Trachurus trachurus,* were found to be similar, for both molecular sequences and morphology, to those found on *Boops boops*. We can therefore confirm, for the first time with molecular evidence, that *B. boops* is a host of this parasite. We consider that *Pseudaxinoides* was erected on the basis of an erroneous interpretation of structures which are not vaginae and, consequently, propose the transfer of most of its species to *Pseudaxine,* as *P. australis* (Lebedev, 1968) n. comb., *P. bychowskyi* (Lebedev, 1977) n. comb., *P. caballeroi* (Lebedev, 1977) n. comb., *P. cariacoensis* (Nasir & Fuentes-Zambrano, 1983) n. comb., and *P. vietnamensis* (Lebedev, Parukhin & Roitman, 1970) n. comb. We also propose *Allogastrocotyle dillonhargisorum* nom. nov. for *Pseudaxine bivaginalis* Dillon & Hargis, 1965 to avoid a secondary homonymy.

## Abstract (fr)

La présence/absence et le nombre de vagins est une caractéristique majeure de la systématique des Monogènes. Trois genres de Gastrocotylidae partagent une morphologie et une anatomie similaires mais se distinguent par ce caractère : *Pseudaxine* Parona & Perugia, 1890 n’a pas de vagin, *Allogastrocotyle* Nasir & Fuentes Zambrano, 1983 a deux vagins et *Pseudaxinoides* Lebedev, 1968 a plusieurs vagins. Au cours d’une étude de *Pseudaxine trachuri* Parona & Perugia 1890, nous avons trouvé des spécimens avec des structures ressemblant à des « vagins multiples ». Nous les avons comparés, pour la morphologie et les molécules (code à barres COI), à des spécimens sans vagin et avons constaté qu’ils appartenaient à la même espèce. Nous avons également examiné l’organe copulateur mâle (OCM) de cette espèce, dont l’exactitude de la description originale est connue pour être un sujet de débat. Nous avons constaté que l’atrium génital est armé de 12 crochets disposés en un seul cercle et d’un stylet central creux qui est probablement impliqué dans l’insémination traumatique. Nous avons redécrit *Pseudaxine trachuri* à partir de spécimens nouvellement collectés des côtes algériennes et de spécimens de Musée des côtes de France. Les spécimens de l’hôte type, *Trachurus trachurus*, se sont avérés similaires, pour les molécules et la morphologie, à ceux trouvés sur *Boops boops*. Nous pouvons donc confirmer, pour la première fois avec des preuves moléculaires, que *B. boops* est un hôte de ce parasite. Nous considérons que *Pseudaxinoides* a été érigé sur la base d’une interprétation erronée de structures qui ne sont pas des vagins et, par conséquent, proposons le transfert de la plupart de ses espèces à *Pseudaxine*, comme *P. australis* (Lebedev, 1968) n. comb., *P. bychowskyi* (Lebedev, 1977) n. comb., *P. caballeroi* (Lebedev, 1977) n. comb., *P. cariacoensis* (Nasir et Fuentes-Zambrano, 1983) n. comb. et *P. vietnamensis* (Lebedev, Parukhin & Roitman, 1970) n. comb. Nous proposons également *Allogastrocotyle dillonhargisorum* nom. nov. pour *Pseudaxine bivaginalis* Dillon & Hargis, 1965, pour éviter une homonymie secondaire.

## Introduction

*Pseudaxine* Parona & Perugia, 1890, *Allogastrocotyle* Nasir & Fuentes Zambrano, 1983 and *Pseudaxinoides* Lebedev, 1968 are three genera that belong to the family Gastrocotylidae Price, 1943. Species of these three genera share a general boot-shaped appearance, because of their oblique haptor, and differ mainly in the presence and number of vaginae. The organisation of internal organs is similar, especially the testes and the male copulatory organ (MCO); however, members of *Pseudaxine* have no vagina, the single member of *Allogastrocotyle* has two vaginae, and species of *Pseudaxinoides* are said to have multiple ventro-lateral vaginae [49, 60, 63, 66].

*Pseudaxine* was established with *Pseudaxine trachuri* Parona & Perugia, 1890, from the gills of *Trachurus trachurus* off Genoa, Italy, as the type species [63]. This species was redescribed several times [53, 64, 67, 70, 76, 77]. One confusing aspect of this parasite was the armature of the genital atrium, which was described as “consisting of *two* crowns of hooks” [63], whereas a single crown was observed in subsequent redescriptions.

During an ongoing investigation on monogeneans infesting the gills of teleosts off the Algerian coast, we collected gastrocotylids consistent with the diagnosis of *P. trachuri* from gills of the type-host, the carangid *T. trachurus*, and also from a sparid, *Boops boops*. Specimens from both hosts were submitted to molecular analysis. Based on this material, we provide a detailed study of the genital atrium of *P. trachuri* and describe for the first time in this species a central stylet.

Unexpectedly, we found several specimens with multiple ventro-lateral papillae, without ducts, which mimicked vaginae. According to the diagnostic criteria of gastrocotylid genera [53], these specimens of *Pseudaxine trachuri* should have been attributed to *Pseudaxinoides*. We compared measurements and COI sequences of these specimens with “multiple vaginae” with typical specimens of *Pseudaxine trachuri* without vaginae, and found that they belonged to the same species. This clearly questioned the alleged differences between *Pseudaxine* and *Pseudaxinoides*, and we finally propose a series of nomenclatural decisions, including the demise of *Pseudaxinoides*.

This work is part of a general survey of the monogenean fauna of fishes of the southern shores of the Mediterranean Sea [5, 7–10, 15–18, 22, 44].

## Materials and methods

### Fishes

During the years 2016 and 2019, 2004 specimens of *B. boops* and 309 *T. trachurus* were collected from off Zemmouri El Bahri (36°48′4.582033″ N, 3°34′7.01″ E), Bouharoun (36°37′24″ N, 2°39′17″ E), Cherchell (36°36′31″ N, 2°11′50″ E), Dellys (36°54′48″ N, 3°54′51″ E), and Cap Djinet (36°52′37″ N, 3°43′23″ E) off the Algerian coast. Fish specimens were purchased dead from fishermen, transferred to the laboratory shortly after capture, and identified using keys [26]. Gills were removed from each fish and observed under a stereo microscope for the presence of monogeneans.

### Monogeneans

#### Morphological methods

Monogeneans were removed from gills using fine dissection needles, killed in hot seawater, then preserved in 70% ethanol, stained with acetic carmine, dehydrated in ethanol series (70, 96 and 100%), cleared in clove oil, and finally mounted in Canada balsam. Drawings were made with the help of an Olympus BH-2 microscope equipped with Differential Interference Contrast (DIC) and a drawing tube. Micrographs were taken with a Leitz microscope. Drawings were scanned and redrawn on a computer with Adobe Illustrator (CS5). Measurements are in micrometres, and indicated as the means, ± the standard deviation if *n* > 30, and in parentheses, the range and number of measurements.

#### Molecular methods

To ensure that hosts and parasites were labelled with respect to their host-parasites relationships, we followed recent works dealing with description of new species of Monogenea by using barcoding [5, 8–10, 43]. For each host fish from which monogeneans were collected, a tissue sample from the gill was taken. Monogeneans were cut into three parts using a scalpel blade. The anterior part (which includes the genital atrium) and posterior part (which includes the haptor) of each specimen were mounted as vouchers (both parts on a single slide) for drawing and deposition in a Museum; their middle part was preserved in absolute ethanol then subjected to molecular analyses. For some specimens, we did not cut them in three part but rather took a lateral part in the middle of the body for the molecular analysis and mounted the rest of the specimen in one part as voucher. Nine specimens were analysed: 2 from the type-host *T. trachurus* (already published in our study of *Allogastrocotyle* [10]; 3 typical specimens from the sparid host *B. boops*, and 4 specimens with “multiple ventro-lateral vaginae” from *B. boops*.

### Molecular barcoding of fish

Total genomic DNA was isolated using a QIAamp DNA Mini Kit (Qiagen, Courtaboeuf, France), as per the manufacturer’s instructions. The 5′ region of the mitochondrial cytochrome *c* oxidase subunit I (COI) gene was amplified with the primers FishF1 (5′ – TCAACCAACCACAAAGACATTGGCAC – 3′) and FishR1 (5′ – TAGACTTCTGGGTGGCCAAAGAATCA – 3′) [75]. PCR reactions were performed in 20 μL, containing 1 ng of DNA, 1× CoralLoad PCR buffer, 3 mM MgCl_2_, 66 μM of each dNTP, 0.15 μM of each primer, and 0.5 units of Taq DNA polymerase (Qiagen). The amplification protocol was 4 min at 94 °C, followed by 40 cycles at 94 °C for 30 s, 48 °C for 40 s, and 72 °C for 50 s, with a final extension at 72 °C for 7 min. PCR products were purified (Ampure XP Kit, Beckman Coulter) and sequenced in both directions on a 3730 × l DNA Analyzer 96-capillary sequencer (Applied Biosystems, Foster City, CA, USA). We used CodonCode Aligner version 3.7.1 software (Codon Code Corporation, Dedham, MA, USA) to edit sequences, compared them to the GenBank database content with BLAST, and deposited them in GenBank under accession numbers MT666082–MT666086. Species identification was confirmed with the BOLD identification engine [68].

### COI sequences of monogeneans

Total genomic DNA was isolated using a QIAmp DNA Micro Kit (Qiagen). The specific primers JB3 (=COIASmit1) (forward 5′ – TTTTTTGGGCATCCTGAGGTTTAT – 3′) and JB4.5 (=COI-ASmit2) (reverse 5′ – TAAAGAAAGAACATAATGAAAATG – 3′) were used to amplify a fragment of 402 bp of the COI gene [11, 55]. PCR reaction was performed in 20 μL of a mixture containing 1 ng of DNA, 1× CoralLoad PCR buffer, 3 mM MgCl_2_, 0.25 mM dNTP, 0.15 μM of each primer, and 0.5 units of Taq DNA polymerase (Qiagen). Thermocycles consisted of an initial denaturation step at 94 °C for 2 min, followed by 37 cycles of denaturation at 94 °C for 30 s, annealing at 48 °C for 40 s, and extension at 72 °C for 50 s. The final extension was conducted at 72 °C for 5 min. PCR products were purified (Ampure XP Kit, Beckman Coulter) and sequenced in both directions on a 3730 × l DNA Analyzer 96-capillary sequencer (Applied Biosystems, Foster City, CA, USA). We used CodonCode Aligner version 3.7.1 software (Codon Code Corporation, Dedham, MA, USA) to edit sequences, compared them to the GenBank database content with BLAST, and deposited them in GenBank under accession numbers MT666075–MT666081 (Table 1).

**Table 1:** Fish, monogeneans and their COI sequences. To insure full traceability and ensure accurate host-parasite relationships, one or several monogeneans were collected from one fish and all individuals were sequenced. All monogeneans in this Table were identified as *Pseudaxine trachuri*.

Fish species	Fish ID	Fish COI sequence	Monogenean ID	Monogenean COI sequence	Voucher deposited in the MNHN
*Boops boops*	Bobo Br1	MT666082	Bobo Br1 MO-01	MT666075	HEL1270
*Boops boops*	Bobo Br1	MT666082	Bobo Br1 MO-02	MT666076	HEL1271
*Boops boops*	Bobo Br1	MT666082	Bobo Br1 MO-03	MT666077	HEL1272
*Boops boops*	Bobo Br2	MT666083	–	–	–
*Boops boops*	Bobo Br4	–	Bobo Br4 MO-01	MT666078	HEL1273
*Boops boops*	Bobo Br7	MT666084	Bobo Br7 MO-01	MT666079	HEL1274
*Boops boops*	Bobo Br8	MT666085	Bobo Br8 MO-01	MT666080	HEL1275
*Boops boops*	Bobo Br9	MT666086	Bobo Br9 MO-01	MT666081	HEL1276

### Trees and distances

Most gastrocotylid sequences available in GenBank were included in a preliminary phylogenetic analysis, with 9 sequences of *Pseudaxine trachuri*, and sequences of two members of the Gotocotylidae Yamaguti, 1963 used as the outgroup. The trees were inferred by the Neighbour-Joining method and the Maximum Likelihood method using MEGA7 [48]. After examining trees and sequences, we refined our analysis and finally used only 12 sequences, as detailed in the results. For the final analysis, we employed the Neighbour-Joining method and the Maximum Likelihood method using MEGA7 [48]; after selecting the best model with MEGA7, the ML was performed with HKY + G [34]. Genetic distances (*p*-distance and Kimura-2 parameter distance [45]) were estimated with MEGA7 and all codon positions were used.

## Results

### Molecular identification of fish

The provisional identification of the fish species using morphological characteristics was confirmed by the DNA barcoding approach. BLAST analysis of the COI sequences of the fish species of the present study with the NCBI and BOLD databases showed sequence similarity values of 100% for *B. boops* and 99.85% for *T. trachurus*.

### Molecular characterization of monogeneans

A preliminary dataset involved 20 COI sequences, including 9 sequences of *P. trachuri* and sequences from GenBank. After alignment and trimming, the dataset was 391 bp in length with 211 informative sites. The main result for this preliminary analysis was that our sequences of *P. trachuri* from off the Algerian coast formed a robust monophylum with the single sequence of *P. trachuri* available from the type-host *T. trachurus* off Sète, France (AY009168) [40]; this sequence is therefore confirmed as *P. trachuri*. However, we noted that sequence AY009167, also from *T. trachurus* off Sète, France, grouped with *P. trachuri*, although it was identified as *Gastrocotyle trachuri* Van Beneden & Hesse, 1863 and labelled as such in GenBank by its authors, Jovelin & Justine [40]; we interpret this as a misidentification, and sequence AY009167 should be considered *P. trachuri*.

**Figure 1:**
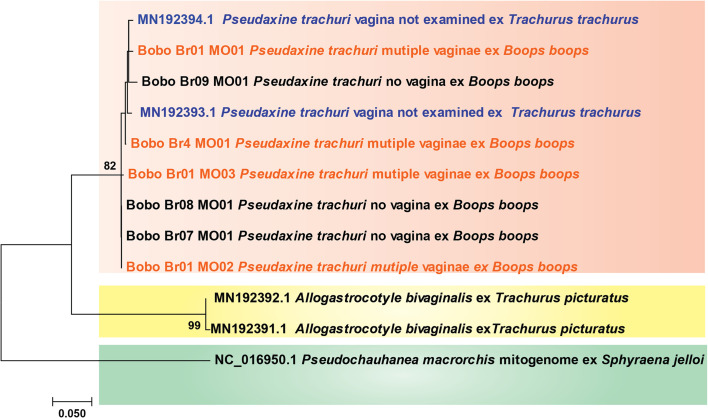
Molecular phylogenetic analysis of monogenean COI sequences using the Maximum Likelihood method. Bootstraps percentages (1000 replicates) are indicated next to the branches when higher than 70. There were a total of 389 positions in the final dataset. The Neighbour-Joining tree had a similar topology and is not represented. All specimens of *Pseudaxine trachuri*, whether with no vagina or with “multiple vaginae”, and whether from the type-host *Trachurus trachurus* or from *Boops boops*, were grouped into a single monophylum and showed little variation (<2%).

We noticed that several GenBank sequences were unconfirmed and/or unpublished, and there was no certainty in the systematic assignment for some of them (in addition to the obvious error mentioned above); also, some had apparent insertions-deletions, or ambiguous bases, and the alignment included gaps. Therefore, from the preliminary dataset, we selected only the sequences that had the same span as our sequences and no ambiguous bases. The restricted dataset included only 12 sequences: 9 sequences of *P. trachuri* (including 7 new), 2 sequences of *Allogastrocotyle bivaginalis*, and the COI part of the complete mitogenome (NC016950) of the chauhaneid *Pseudochauhanea macrorchis* Lin, Liu & Zhang in Zhang, Yang & Liu, 2001 [81]. We obtained perfect alignment, 389 bp in length, without any gap or missing data. The tree (Fig. 1) showed a robust monophylum that includes all sequences of *P. trachuri*. The *P. trachuri* clade contained sequences from the type-host and from the sparid host *B. boops*, confirming the occurrence of *P. trachuri* on a sparid. Moreover, all specimens of *P. trachuri* with apparent “multiple ventro-lateral vaginae” and specimens without vaginae clustered in the same clade, without having a distinct branch.

Distances were computed using p-distance and the Kimura-2 parameter. All sequences of *P. trachuri*, whatever host or apparent number of vaginae, were very similar, with less than 2% variation. This suggests that *P. trachuri* also occurs on *B. boops* and that specimens with apparent multiple vaginae are conspecific with typical *P. trachuri*.

### Morphology of *Pseudaxine trachuri* Parona & Perugia, 1890 (Figs. 2–4)

*Type-host: Trachurus trachurus* (Linnaeus, 1758) (Carangidae), Atlantic horse mackerel.

**Figure 2:**
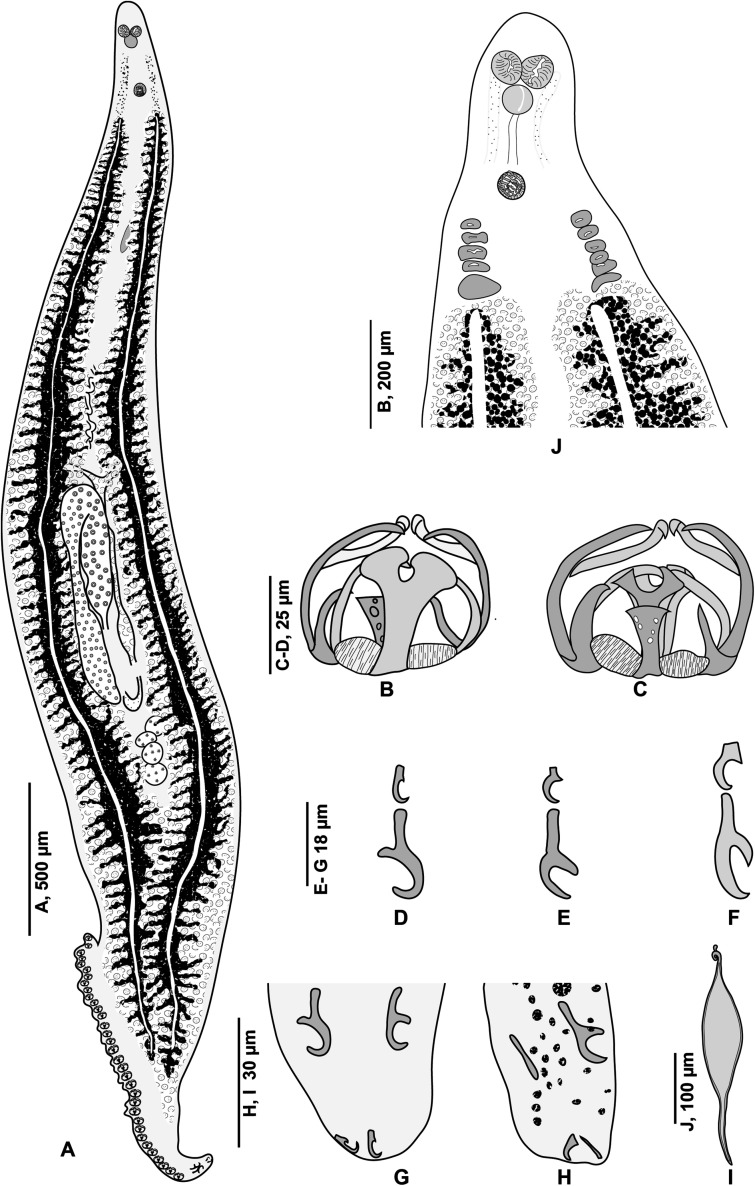
*Pseudaxine trachuri* Parona & Perugia, 1890. (A) Entire worm, typical specimen without a vagina, MNHN HEL1278; (B) clamp, ventral view; (C) clamp, dorsal view, MNHN HEL1278; (D–F) anchors from terminal lappet, MNHN HEL1279, HEL1280; (G and H), terminal lappet, MNHN HEL1280, HEL1281; (I) egg, MNHN HEL1282. (J) anterior extremity of a specimen from *Boops boops*, showing apparent “multiple lateral vaginae” (MNHN HEL1283).

**Figure 3:**
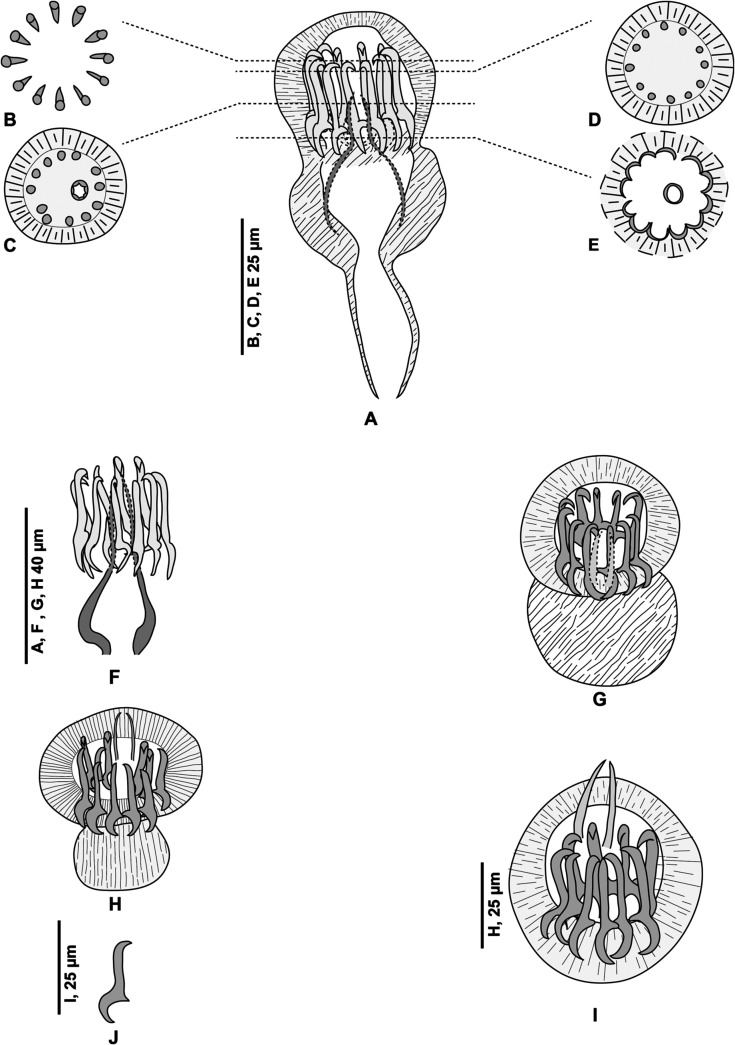
*Pseudaxine trachuri*, genital atrium shown laterally and in different optical sections and the variable position of the stylet. (A) Genital atrium, lateral view, MNHN HEL1284; (B–E), optical sections of a genital atrium in perfect apical view; the level of these sections are shown on the lateral view, MNHN HEL1285. (F) Lateral view of the atrium showing only the sclerotised parts, MNHN 306HG, Box 43, slide 51; (G–I) various views at different orientations, MNHN HEL1286, MNHN HEL1287, MNHN HEL1288. (J) One spine, MNHN HEL1287.

**Figure 4:**
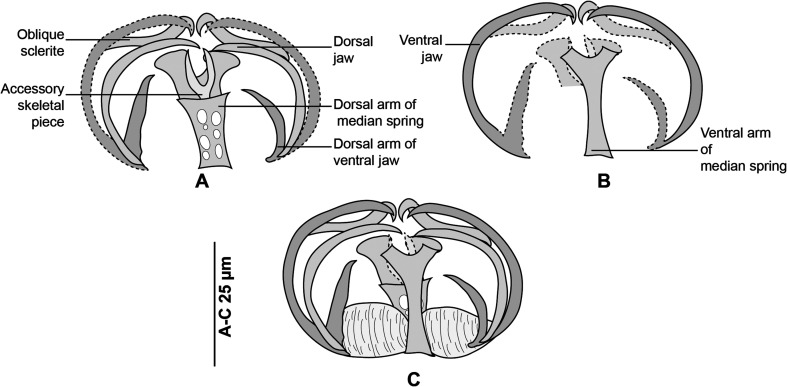
Clamp, with detailed legends, MNHN HEL1289. (A) Ventral jaw. (B) Dorsal jaw. (C) Whole clamp.

*Type-locality:* Off Genoa (Genova), Italy.

*Additional host: Boops boops* (Linnaeus, 1758) (Sparidae), bogue.

*Site on host:* Gills.

*Additional localities*: off Zemmouri el Bahri (36°48′4.58″ N, 3°34′7.01″ E), Bouharoun (36°37′24″ N, 2°39′17″ E), Cherchell (36°36′31″ N, 2°11′50″ E), Dellys (36°54′48″ N, 3°54′51″ E) and Cap Djinet (36°52′37″ N, 3°43′23″ E), off the Algerian coast (present study).

*Prevalence and intensity*: in *T. trachurus* off Algeria: 28% (89/309), up to 3 monogeneans/fish; in *B. boops* off Algeria: 10% (201/2004), up to 2 monogeneans/fish.

*Specimens from Algerian waters studied and deposited*: 14 vouchers from the type-host, *T. trachurus*, including 2 with molecular information (MNHN HEL1093, GenBank MN192393, MNHN HEL1094, GenBank MN192394); 23 specimens from *B. boops* (HEL1270–HEL1292), including 7 with molecular information (HEL1270–HEL1276, GenBank MT666075–MT666081, Table 1). Vouchers with molecular information consist of the anterior and posterior parts of specimens mounted together on a slide; while the middle part was used for molecular analysis.

*Comparative material studied*: 6 specimens of *P. trachuri* from its type-host *T. trachurus* off Sète, French Mediterranean Coast; part of the collection of Prof. Louis Euzet deposited in the Muséum National d’Histoire Naturelle (MNHN 306HG, Box 43, slides 19, 51, 54, 55–63, 80, 81).

#### Description of specimens from *Boops boops* from off Algeria

Based on 30 specimens. Measurements in Table 2. Body elongate, typically gastrocotylid. Haptor, oblique, asymmetrical, with single row of clamps. Clamps typically gastrocotylid; ventral arm of median spring Y-shaped; proximal end of ventral arm of median spring narrow; its distal part Y-shaped; dorsal arm of median spring short, has 3–4 pairs of apertures arranged in 2 longitudinal, symmetrical, parallel rows; distal end of dorsal arm with accessory skeletal piece represented by V-shaped process at its distal end (Fig. 4). Dorsal arm of ventral jaw short and curved inwards. Dorsal jaw shorter than ventral. Oblique sclerites with inner ends touching each other in median line. Base of clamp with cuticularised hinge ligament on each side, connecting median spring with base of jaw sclerites (Fig. 4).

**Table 2:** Measurements of *Pseudaxine trachuri* from different hosts and localities.

Source	Parona & Perugia [63]	Yamaguti [76]	Yamaguti [77]	Sproston [70]	Radujkovic & Euzet [67]	Present study	Present study
Host	*T. trachurus*	*T. trachurus*	*T. trachurus*	*T. trachurus*	*T. mediterraneus*	*T. trachurus*	*B. boops*
Locality	Italy, M.S.	Japan, P.O.	Japan, P.O.	Plymouth, N.A.	Montenegro, M.S.	Algeria, M.S.	Algeria, M.S.
Haptor length						784 (560–1440, *n* = 12)	954 (500–1400, *n* = 27)
Total length	4000–6000	2700–3100	3000	2300	5000–6000	2836 (2250–4100, *n* = 14)	2400 ± 482 (1450–3300, *n* = 31)
Total width		360–400		530	200–250	583 (310–870, *n* = 14)	647 ± 150 (300–900, *n* = 31)
Number of clamps	24–32	20–22	31	27	24–32	25 (17–30, *n* = 15)	27 ± 2 (20–30, *n* = 34)
Clamps length						43 (31–50, *n* = 28)	40 (30–48, *n* = 29)
Clamps width	60			51		30 (20–50, *n* = 28)	26 (20–34, *n* = 29)
Distal pair length	25	24	33	23	25	27 (21–30, *n* = 13)	29 (24–36, *n* = 27)
Proximal pair length	10	12	15	13.5		10 (6–12, *n* = 11)	13 (12–14, *n* = 20)
Buccal organ length	40*	36–42	39			34 (20–50, *n* = 12)	39 ± 5 (25–48, *n* = 35)
Buccal organ width		33–36	33			34 (20–50, *n* = 12)	38 ± 5 (25–48, *n* = 35)
Pharynx length		42	45			45 (28–60, *n* = 10)	42 ± 5 (31–50, *n* = 33)
Pharynx width		36	36			44 (28–55, *n* = 10)	41 ± 5 (28–50, *n* = 33)
Atrium length						39 ± 7 (25–48, *n* = 14)	34 ± 4 (28–45, *n* = 34)
Atrium width						42 ± 4 (35–48, *n* = 14)	34 ± 4 (28–45, *n* = 34)
Number of atrial hooks	>24	13	14	16	12	12 ± 0 (12–12, *n* = 13)	12 ± 0 (12–12, *n* = 31)
Genital hooks length		20	20			21 ± 1 (20–21, *n* = 16)	17 (12–20, *n* = 26)
Distance genital atrium to anterior extremity		300–330	300			238 (180–370, *n* = 11)	229 ± 48 (130–360, *n* = 33)

* Diameter.

Areas: M.S., Mediterranean Sea; N.A., North Atlantic; P.O., Pacific Ocean.

Terminal lappet present, armed with 2 pairs of anchors, dissimilar in shape and size (Figs. 2D–2F); anteriormost anchor pair large, situated at base of haptoral lappet; posteriormost anchor pair small, at tip of haptoral lappet (Figs. 2G–2H). No additional pair observed.

Pair of prohaptoral suckers sub-oval, muscular and septate. Pharynx pyriform. Intestinal caeca extend to posterior end of body, not confluent (Fig. 2A).

Testes postovarian, large and oval, arranged irregularly in intercaecal field in posterior third of body. Vas deferens passes forward along body midline and enlarges to form ejaculatory bulb at level just anterior to genital atrium (Figs. 3A, 3G and 3H).

Ovary pretesticular, in form of inverted “U”. Ovary begins at level of anteriormost testes, continues anteriorly in midline, reflexes and passes posteriorly toward haptor to terminate as oviduct. Oviduct arises from distal end of ovary and opens into oötype with Mehlis’ gland cells. Uterus, arises from oötype and passes anteriorly. Genito-intestinal canal short, ventral to ovary. Vitellarium follicular, co-extensive with intestinal caeca, extending from level of genital atrium to haptor. Transverse vitelloducts fuse in midline, ventral to ovary, to form Y-shaped vitelline reservoir. Vagina absent (Fig. 2J).

##### Genital atrium

Genital atrium ventral, with a muscular rim, located in the anterior constriction of the body. The genital atrium includes 2 sclerotised structures: a crown of 12 hooks and a central stylet. Gastrocotylid-like atrial hooks (Fig. 3J) are arranged as a perfect circle; this was seen in some specimens in which the hooks are aligned with the optical axis of the microscope, i.e. perpendicular to the longitudinal body axis. In these polar views, observation with a DIC microscope, which has a very low field depth, enables various optical sections, from distal to proximal: the distal (external) extremity of the hooks, with their points directed toward the centre; the middle part, with cross-sections of cylindrical hooks in circles; and basal region, with the blades of hooks forming a continuous circle. Observation of genital atria with a longitudinal orientation of the hooks confirms this morphology, with the pointed ends directed toward the centre and blade-like bases. The central stylet is a slender, conical structure, which appears in cross-sections as a hollow circle (Figs. 3C and 3E) and in longitudinal sections as a conical, pointed structure (Figs. 3A and 3F–3I). The position of the stylet is variable in respect to the crown of hooks and can protrude distally from it (Figs. 3H and 3I) or not (Figs. 3A, 3F and 3G). The stylet is connected basally with an unsclerotised bulb, which is itself connected at its base to the vas deferens (Fig. 3A). In flattened whole-mounts, the stylet is occasionally tilted such that it appears as a pair of slender spines (Fig. 3I).

##### Vagina and multiple pseudo-vaginae

In most specimens, a vagina was absent; the DIC microscope enables one to examine with precision the surface of the tegument and we did not detect any opening. In a series of specimens from *B. boops*, we found, however, multiple ventro-lateral papillae which could be interpreted as two rows of lateral vaginae. We tried to find a duct leading from these openings to the posterior parts, but did not detect it; instead, we noticed that flame cells were often close to these apertures. We consider, therefore, that these openings are pores of the excretory system, which mimic lateral vaginae.

**Figure 5:**
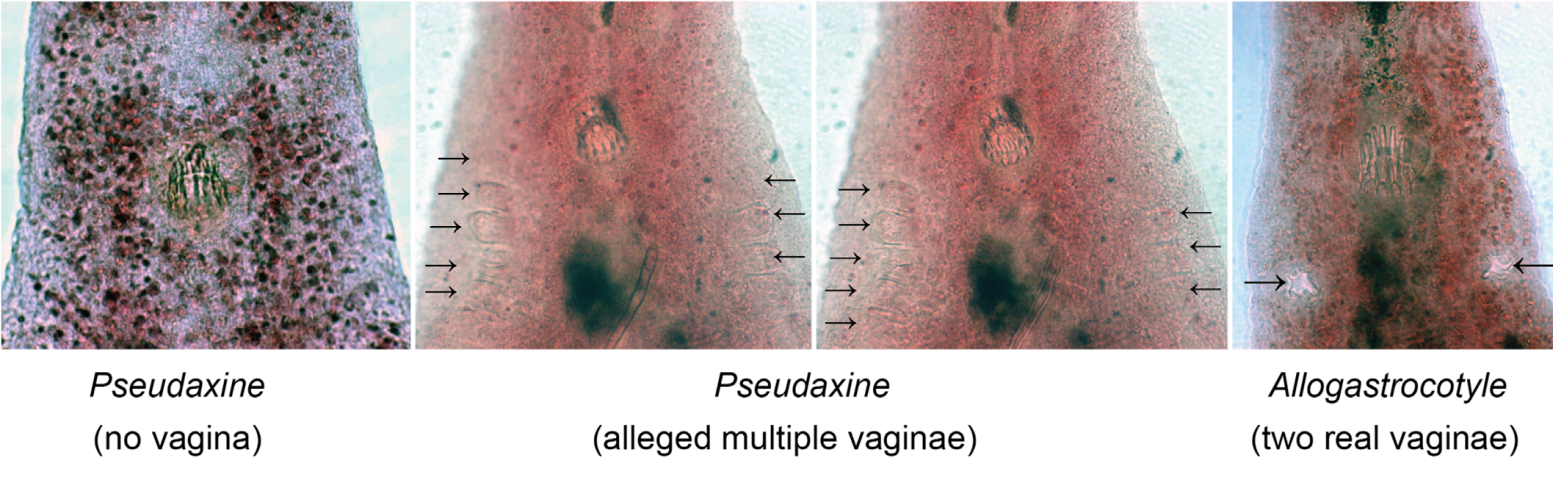
Micrographs of specimens showing the region of the genital atrium and vaginae (arrows). Left, *Pseudaxine trachuri*, no vagina visible; Middle, *Pseudaxine trachuri*, alleged multiple vaginae, two different focuses on the same specimen; Right, *Allogastrocotyle bivaginalis*, two real vaginae. Unscaled, same scale for all micrographs.

Figure 5 shows micrographs of the region of the genital atrium and vaginae in *P. trachuri* (both specimens without a vagina and with alleged multiple vaginae) and in *Allogastrocotyle bivaginalis*.

## Discussion

### *Boops boops* as a host for *Pseudaxine trachuri*

*Pseudaxine trachuri* is a cosmopolitan monogenean, reported from many carangid hosts, mainly *Trachurus* spp., and from a single sparid, *Boops boops* (Table 3). In the present study, measurements and counts of *P. trachuri* from *B. boops* were within range of previous descriptions (Table 2), and COI sequences of *P. trachuri* from *B. boops* differed from those from the type-host by only 1–2%. Differences higher than 4% in COI sequences are generally interpreted as an indication that species are different [4, 33, 74, 75]; in polyopisthocotylean monogeneans, differences in COI sequences reported as intraspecific ranged 0.2–5.6% [8]. The 1–2% difference reported here is well within the intraspecific range. We thus confirm, for the first time with molecular evidence, that *P. trachuri* uses *B. boops* as a host. We noted that, in our specimens, the prevalence in *B. boops* was lower than in *T. trachurus* (10% vs. 28%), which could be an indication of a lower adaptation to the sparid host, or a recent host-switch.

**Table 3:** Hosts and localities of *Pseudaxine trachuri* from the literature. Order: type-host first, then other species of *Trachurus*, then other species in alphabetical order. For *Boops boops*, the compilation by Pérez-del Olmo [65] includes many previous records.

Host, locality	References
Carangidae	
*Trachurus trachurus*	
Mediterranean	[24, 37, 40, 47, 63, 69], present study
Atlantic	[3, 14, 27, 28, 70]
Pacific	[64, 76, 77]
*Trachurus capensis*	
Atlantic	[27, 28]
Pacific	[64]
*Trachurus declivis*	
Pacific	[46]
*Trachurus lathami*	
Atlantic	[2, 13]
*Trachurus mediterraneus*	
Mediterranean	[1, 19, 59, 61, 67, 71]
*Trachurus novaezelandiae*	
Pacific	[49]
*Trachurus picturatus*	
Mediterranean	[32]
Atlantic	[20, 27, 29, 35]
*Carangoides malabaricus* (as *Caranx malabaricus), Caranx* sp., *Decapterus* sp. *Carangoides* sp.	
Pacific	[50, 64]
*Decapterus muroadsi*	
Pacific	[30]
Sparidae	
*Boops boops*	
Mediterranean	[6, 25, 38, 58, 62, 65], present study
Atlantic	[65]

### General structure of the genital atrium of *Pseudaxine trachuri*

The original description of *P. trachuri* is apparently very detailed and beautifully illustrated, especially for a paper of the 19th Century [63]. It has been supplemented by redescriptions and more morphometric data by Yamaguti [76, 77], Sproston [70], Parukhin [64], Lebedev [53] and Radujkovic & Euzet [67]. However, the illustrations were sometimes poor or redrawn from the original 1890 account of Parona & Perugia [63].

Parona and Perugia indicated that the genital atrium was armed with two crowns of hooks [63]: “*L’apertura cloacale […] coronata da un cerchio di 24 uncini minutissimi (Fig. 11), con base allargata e punta arcuata rivolta verso il lume del canale. Inferiormente e nell’interno della cloaca si osserva altro cerchio di aculei (Fig. 12) molto più lunghi dei superiori (0,017) che soprastanno all’apertura del deferente e che si può rigardare quale armatura maschile*” which translates as “The cloacal opening […] is crowned by a circle of 24 very fine hooks (Fig. 11), with an enlarged base and the point turned towards the lumen of the canal. Beneath and inside the canal, another circle of spines is observed (Fig. 12), which are much longer than those above (0.017) and are present distal to the aperture of the vas deferens; these can be considered the male armature”.

Yamaguti redescribed the species twice, on the basis of two (1938) and one (1942) specimens, from the gills of the type-host off Japan [76, 77]. In both descriptions, Yamaguti clearly indicated that the genital atrium was surrounded by a single crown of hooks, but did not comment on the differences in the genital atrium.

Sproston (1946) redescribed *P. trachuri* from its type-host, based on a single specimen collected from off Plymouth, England, and indicated the presence of 16 atrial hooks. However, she reproduced in her paper one of the drawings by Parona & Perugia and commented on the original description: “these authors give a curious figure of the genital corona […] and describe it as having an outer ring of 24 small hooks and 17 larger hooks below at the end of the vas deferens”; she stated “neither the Japanese nor Plymouth specimens from the same host reveal anything like this structure”. In this sentence, Sproston mentioned 17 larger hooks, but Parona & Perugia did not provide the number of the larger hooks; a possible explanation is that Sproston confused the length (17 μm) and the number of the larger hooks in the original description in Italian, although the length was provided as “0.017”.

Unnithan [73], probably using Sproston’s text, wrote in his description of *Pseudaxine kurra* Unnithan, 1968 “no structure identical to the genital corona having an outer rim of 24 small hooks and 17 larger hooks below it at the end of the vas deferens is observed in the new species”.

Lebedev commented “The discussion of Sproston [70] and Unnithan [73] about the allegedly incorrect description of these structures by Parona and Perugia [63] seems unreasonable to us: if we accept that the outer ring of spines in the picture of the copulatory apparatus given in the first description is the apical parts of the hooks, and the inner ring is a stylet, then everything falls into place [53].

Radujkovic & Euzet, on the basis of specimens from off Montenegro, described briefly the genital atrium as having a crown of 12 spines [67].

Our careful examination of new specimens of *Pseudaxine trachuri* from different hosts and localities showed the presence of a single crown (corona) of 12 hooks. Clearly, the two circles described by Parona & Perugia [63] have never been mentioned in all subsequent descriptions of this species (Table 4). Although Lebedev [53] suggested that this could be considered a minor misinterpretation of the structure, we believe that it is important to clarify this point.

**Table 4:** Descriptions of the genital atrium of *Pseudaxine trachuri*.

Reference	Host	Locality	Description of genital atrium
Parona & Perugia [63]	*T. trachurus*	Off Italy, M.S.	Circle of 24 very fine hooks, with enlarged base and point turned towards lumen plus
			Second circle of longer hooks (17 μm), above aperture of vas deferens
Yamaguti [76]	*T. trachurus*	Off Japan, P.O.	Crown of 13 hooks, each of which is about 20 μm long and has long blade with it tips strongly curved posteriorly and inwards, and with thickening near its base, by which it connects with neighbouring hooks
Yamaguti [77]	*T. trachurus*	Off Japan, P.O.	Ring of 14 hooks about 20 μm long
Sproston [70]	*T. trachurus*	Off Plymouth, N.A.	Ring of 16 hooks
Radujkovic & Euzet [67]	*T. mediterraneus*	Off Montenegro, M.S.	Crown of 12 spines (20 μm)
Present study Collection of Pr. Euzet, MNHN	*T. trachurus*	Off France, M.S.	Crown of 12 hooks and central stylet
Present study	*T. trachurus, B. boops*	Off Algeria, M.S.	Crown of 12 hooks and central stylet

Areas: M.S., Mediterranean Sea; N.A., North Atlantic; P.O., Pacific Ocean.

The number of atrial hooks in the material examined in this study and other accounts (Table 2) varies according to locality. Yamaguti suggested that it was due to intraspecific variability [76, 77]. However, the number of hooks given in this study is the same as for the specimens from the Mediterranean described by Radujkovic and Euzet [67]. It may be that specimens from seas other than the Mediterranean correspond to different species; this needs to be investigated using molecular methods. We provisionally consider that all records worldwide correspond to one and the same species.

In none of the previous description was a stylet mentioned. Although Lebedev used this term to explain the drawing by Parona & Perugia, he did not explicitly describe a stylet in his specimens.

### The central stylet of the genital atrium

We describe in our specimens of *P. trachuri* the presence of a conical structure in the centre of the crown of spines of the genital atrium; this is the first time that such as structure has been described in this species. Llewellyn (1983) provided a detailed description of the genital atrium of *Gastrocotyle trachuri*; he named the hooks forming the crown “ribs” and the central stylet a “penis tube” [56]; he also proposed a hypothesis for sperm transfer in which the genital atrium would work as a sucker and the penis tube would penetrate the tegument to allow the deposition of sperm in the subtegumental tissue of the partner. What could be a stylet-like structure was also described by George (1960) in another gastrocotylid, *Engraulicola forcipopenis* George, 1960, as a “forceps-like spine at the end of the penis” [31]. In the diclidophorid *Diclidophora merlangi* (Kuhn, 1829) Krøyer, 1838, which has no vagina, MacDonald & Caley showed that sperm were injected into the tissue of the partner [57]; however, a stylet was not described in this species.

Our observations of the structure of the stylet show that it is hollow and thus could allow sperm to be injected into the tissue of the partner, and the absence of a vagina (see below) suggests that *P. trachuri* uses traumatic insemination, a process which is not uncommon in polyopisthocotylean monogeneans [41].

### Vaginae in specimens of the genus *Pseudaxine* and allied genera

Parona & Perugia, in their original description of *P. trachuri*, which is interspersed with comparison with another species, clearly stated that there was no vagina “*Infatti non travammo traccia di canale e di apertura vaginale*”, i.e. “In fact we did not find a trace of a canal or of a vaginal opening” [63].

Yamaguti redescribed the species twice [76, 77]. In neither description did he mention the presence or absence of a vagina. However, in his “Systema Helminthum” he wrote for the diagnosis of *Pseudaxine* “vagina opening middorsally” [78].

Dillon & Hargis described *Pseudaxine bivaginalis* Dillon & Hargis, 1965, and emended Yamaguti’s (1963) diagnosis of the genus to include the presence of “a single vagina opening middorsally or paired vaginae opening near lateral margins at or near level of genital atrium” [23].

Unnithan described *Pseudaxine kurra* Unnithan, 1968, with “vaginal pore unarmed, dorsal, with few gland cells and situated in front of the ovarian loop, slightly shifted to the right side”; apparently unaware of Dillon & Hargis’s work (1965), he emended the diagnosis of the genus as “vagina single median dorsal and unarmed” [73]. Lebedev commented on the position of the vagina in *Pseudaxine kurra*: “Most likely a mistake was made, since the vagina cannot be in this place. In our specimen (not in very good condition) no vagina was found”; and he questioned the validity of the species [53].

Radujkovic & Euzet, for *P. trachuri* from off Montenegro, wrote “the vagina seems absent” [67].

Lebedev recorded *Pseudaxine trachuri* from *Trachurus novaezelandiae* (which is the type-host of *Pseudaxine bivaginalis* Dillon & Hargis, 1965) off Australia in 1968, and mentioned the presence of two lateral vaginal openings [49]. Since two vaginae have never been observed in *P. trachuri* (Table 5), we believe it likely that the gastrocotylids recorded by Lebedev on *T. novaezelandiae* were in fact *P. bivaginalis* (see the taxonomic decision made regarding this taxon below).

**Table 5:** Vaginal opening and its position in some species attributed to *Pseudaxine*.

Species	Vagina	Source
*Pseudaxine trachuri* Parona & Perugia, 1890	Absent/not observed	Parona & Perugia [63] and Radujkovic & Euzet [67]
*Pseudaxine kurra* Unnithan, 1968	Single, mid-dorsal	Unnithan [73]
*Pseudaxine kurra* Unnithan, 1968	Not found	Lebedev [53]
*Pseudaxine bivaginalis* Dillon & Hargis, 1965*	Paired, ventro-lateral, slightly posterior to genital atrium	Dillon & Hargis [23]
*Pseudaxine bivaginalis* * (referred to as *Pseudaxine trachuri*, see discussion)	Paired, postero-lateral to genital atrium	Lebedev [49]
*Pseudaxine bivaginalis* *	Paired, ventro-lateral, close to genital atrium, surrounded by muscular tissue	Lebedev [53]

* We propose herein to reassign this species to *Allogastrocotyle* as *Allogastrocotyle dillonhargisorum* nom. nov.

Yamaguti described *Pseudaxine decapteri* Yamaguti, 1968 with a “vagina on each side divided into longitudinal row of 5–13 transversely elongated cuticular pits, beginning just behind level of genital pore” [80]. He also claimed “The peculiar structure of the vagina must have been overlooked by Parona and Perugia and subsequent authors because it is recognizable in well preserved specimens alone”. Subsequently, Lebedev erected the genus *Pseudaxinoides* Lebedev, 1968 for monogeneans resembling *Pseudaxine* spp. but differing from it by the presence of multiple ventro-lateral vaginal papillae [49]. Lebedev (1977) studied descriptions and figures of *Pseudaxine decapteri* and assigned this species to *Pseudaxinoides* [51].

### Taxonomic decisions

**Figure 6:**
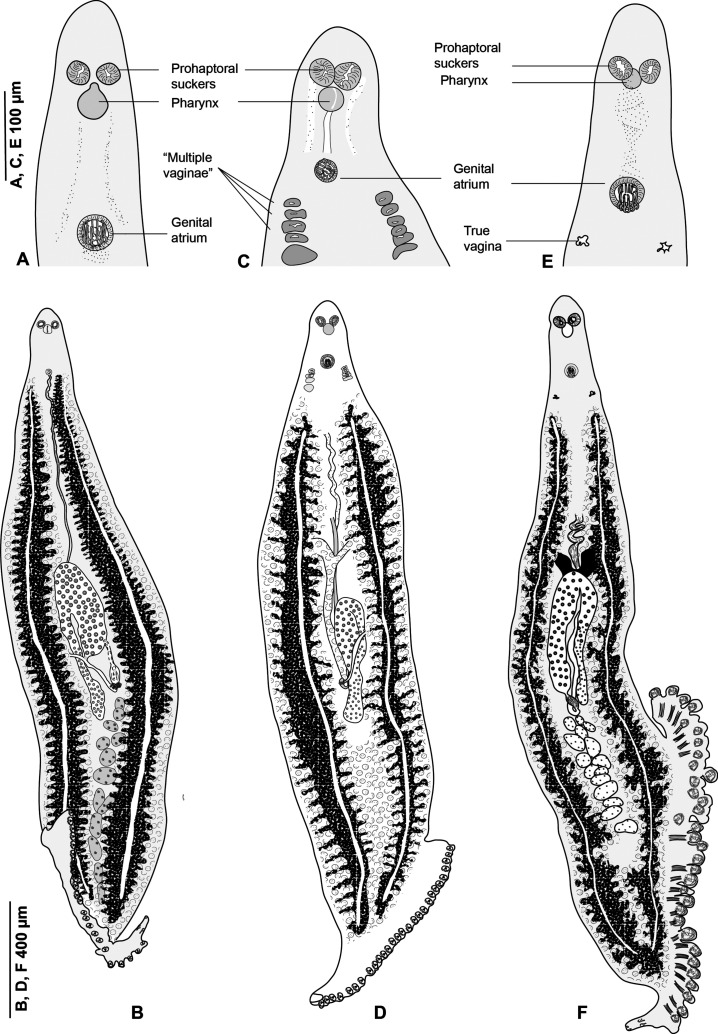
Illustrations of worms with the apparent vaginal arrangement agreeing with the diagnoses of three different genera: *Pseudaxine, Pseudaxinoides,* and *Allogastrocotyle*. (A and B) *Pseudaxine trachuri*, typical aspect, no vagina, MNHN HEL1290, HEL1291; (C and D) *Pseudaxine trachuri*, specimen with apparent “multiple vaginae”, MNHN HEL1283, HEL1292; (E and F), *Allogastrocotyle bivaginalis*, two real vaginae, MNHN HEL1293 (habitus redrawn from Bouguerche et al. [10]).

As explained above, three genera of gastrocotylids are very similar in all aspects, except for their (alleged) number of vaginae. Figure 6 shows drawings of, side by side, specimens of *Pseudaxine trachuri* (no vagina), *Pseudaxine trachuri* (“multiple vaginae”), and *Allogastrocotyle bivaginalis* (two vaginae). Figure 5 shows micrographs of the anterior region of similar specimens. Specimens with multiple vaginae would have been attributed to *Pseudaxinoides* in its traditional concept.

In the present study, we have demonstrated, on the basis of morphology and molecules, that specimens of *P. trachuri* without a vagina and specimens with apparent multiple lateral vaginae are conspecific. We thus consider that all similar species described with apparent multiple vaginae are based on an erroneous interpretation of the excretory system; this makes the distinction between *Pseudaxine* and *Pseudaxinoides* void, and we propose the synonymy of these two genera. Since *Pseudaxine* has priority over *Pseudaxinoides* (1890 vs. 1968), we propose to transfer most *Pseudaxinoides* spp. to *Pseudaxin*e, with the following new combinations: *Pseudaxine australis* (Lebedev, 1968) n. comb.; *Pseudaxine bychowskyi* (Lebedev, 1977) n. comb.; *Pseudaxine caballeroi* (Lebedev, 1977) n. comb.; *Pseudaxine cariacoensis* (Nasir & Fuentes-Zambrano, 1983) n. comb.; and *Pseudaxine vietnamensis* (Lebedev, Parukhin & Roitman, 1970) n. comb. [49–52, 54, 60, 80]. *Pseudaxine decapteri* Yamaguti, 1968 is kept within *Pseudaxine*. The status of *P. kurra* is pending [53]. We noticed that *Pseudaxinoides pusanowi* Lebedev, 1984 has two of its testes that are paraovarian [52], in contrast to species of *Pseudaxine* in which they are all post-ovarian; Lebedev noted later [53] that is was close to species of *Allopseudaxinoides* Yamaguti, 1968; a reappraisal of this species is probably necessary and thus we provisionally refrain from transferring it to *Pseudaxine*.

A comparison of *Pseudaxine bivaginalis* Dillon & Hargis, 1965 with the single species of *Allogastrocotyle, A. bivaginalis* Nasir & Fuentes Zambrano, 1984 [10, 60], suggests that the former species should be transferred to *Allogastrocotyle*, as indicated by the presence of two ventrolateral vaginal openings surrounded by muscular tissue near the genital atrium [23, 53]. Since the name *Allogastrocotyle bivaginalis* is already occupied, we propose here the new name *Allogastrocotyle dillonhargisorum* nom. nov. to avoid homonymy. The new name has been registered in ZooBank as: urn:lsid:zoobank.org:act:1ECC6CF2-4BC4-45E8-9AE7-049C00FC7735.

### Comments on other genera

We noticed that “multiple lateral vaginae” have been described in other polyopisthocotylean monogeneans. Here are a few examples:

- The description of *Sibitrema poonui* Yamaguti, 1966, type-species of *Sibitrema* Yamaguti, 1966, reads as: “vaginae symmetrical, about 0.5 mm long, divided into a series of several (8–14) areolae, situated laterally about halfway between genital pore and anterior end of vitellaria” [79].
- The description of *Allopseudaxine yaito* Yamaguti, 1968 reads as “vaginae opening ventrosubmarginally at a distance of 0.9 mm from head end in the type, each divided into a longitudinal row of about ten transverse slit-like areolae with heavily cuticularized margins” [80].
- The figure of *Gastrocotyle indica* Subhapradha, 1951 in Yamaguti (1963; his Figure 398) shows multiple lateral vaginae, which were commented upon by Lebedev (1986) [53, 72, 78].

Considering that in a single species, *P. trachuri*, we found specimens with and without what look like “multiple lateral vaginae”, we believe that the taxa mentioned above, and probably others, should be re-examined, preferably using both morphology and molecular information. More generally, we stress the importance of integrative taxonomy [21] for the taxonomy of parasites, including correct identification of hosts using both morphology and molecular analysis as in the present work and recent studies [5, 8–10, 43] and, whenever possible, deposition of hosts in curated collections [12, 36, 39, 42].
